# Is Attention Really Effort? Revisiting Daniel Kahneman’s Influential 1973 Book *Attention and Effort*

**DOI:** 10.3389/fpsyg.2018.01133

**Published:** 2018-09-06

**Authors:** Brian Bruya, Yi-Yuan Tang

**Affiliations:** ^1^Department of History and Philosophy, Eastern Michigan University, Ypsilanti, MI, United States; ^2^Department of Psychological Sciences, Texas Tech University, Lubbock, TX, United States; ^3^Center for Advanced Study in the Behavioral Sciences, Stanford University, Stanford, CA, United States

**Keywords:** attention, effort, effortless, LC-NE system, meditation, parasympathetic dominance, pupil dilation, sympathetic dominance

## Abstract

Daniel Kahneman was not the first to suggest that attention and effort are closely associated, but his 1973 book *Attention and Effort*, which claimed that attention can be identified with effort, cemented the association as a research paradigm in the cognitive sciences. Since then, the paradigm has rarely been questioned and appears to have set the research agenda so that it is self-reinforcing. In this article, we retrace Kahneman’s argument to understand its strengths and weaknesses. The central notion of effort is not clearly defined in the book, so we proceed by constructing the most secure inferences we can from Kahneman’s argument regarding effort: it is cognitive, objective, metabolic expenditure, and it is attention. Continuing, we find from Kahneman’s argument that effort-attention must be a special case of sympathetic dominance of the autonomic nervous system that is also an increase in metabolic activity in the brain that has crossed a threshold of magnitude. We then weigh this conception of effort against evidence in Kahneman’s book and against more recent evidence, finding that it does not warrant the conclusion that effort can be equated with attention. In support of an alternative perspective, we briefly review diverse studies of behavior, physiology, and neuroscience on attention and effort, including meditation and studies of the LC-NE system, where we find evidence for the following: (1) Attention seems to be associated not with the utilization of metabolic resources *per se* but with the readying of metabolic resources in the form of adaptive gain modulation. This occurs under sympathetic dominance and can be experienced as effortful. (2) Attention can also occur under parasympathetic dominance, in which case it is likely to be experienced as effortless.

*Any domain of scientific research has its sustaining orthodoxy. That is, research on a problem, whether in astronomy, physics, or biology, is conducted against a backdrop of broadly shared assumptions. It is these assumptions that guide inquiry and provide the canon of what is reasonable—of what “makes sense.” And it is these shared assumptions that constitute a framework for the interpretation of research results.*- Churchland et al. (1994, p. 23)

## Introduction

In 1973, Daniel Kahneman published *Attention and Effort*, which summarized over a decade’s worth of groundbreaking studies on a variety of aspects of attention, including divided attention, task interference, and the role of perception. With these studies, scientists were finally making progress in not just understanding what attention is but in measuring and quantifying it. For example, Kahneman (1973) describes the results of several research programs, including his own, that measured pupil dilation as an index of mental effort. Pupil reactions correlated so closely with what appeared to be the work being done by the brain that it was natural for Kahneman to conclude that pupil reactions indicated both attending to the task and the effort exerted in attending to the task. And so it seemed uncontroversial to conclude that attention is effort.^1^

Although Kahneman examines a large number of studies of attention and weaves evidence from them together into a complex, multi-faceted theory, he overlooks a crucial step in the process of theory-building, namely, defining his operationalized terms. His evidence is also limited largely to laboratory studies that elicit only what may be called *forced attention* from subjects. Subjects are instructed to direct their attention to decontextualized items selected by researchers rather than contextualized items of their own interest, contrary to how attention is directed in ecologically valid circumstances. As a result, it is difficult to conclude that Kahneman offers an adequately operationalized theory of attention.

Despite its limitations, Kahneman’s association of attention and effort has achieved the status of paradigmatic truth. Not only has his book been cited over 10,000 times in the literature, but the basic idea that attention is correlated with effort has influenced research programs to the extent that researchers rarely entertain the idea that attention could be anything but effortful.^2^ While attention as effort may seem intuitive, a moment’s reflection opens the idea to doubt.

Consider attention-deficit/hyperactivity disorder (ADHD). If attention were effort, we could substitute “effort” wherever we see the word “attention.” ADHD could then be EDHD—effort-deficit/hyperactivity disorder—affected people aren’t trying hard enough. Consider also games, sports, hobbies, and other forms of entertainment. We spend a significant portion of our lives paying very close attention to such things, and yet they are often not experienced as effortful but just the opposite—as a break from other activities that require effortful attention.

At the very least, if Kahneman’s theory is correct, it requires some disambiguation to free it from easy refutation by arguments related to the above two considerations. Perhaps Kahneman’s notion of effort is not a notion of trying hard or of a feeling of effort, but both of these are natural construals of the word “effort,” and both can find support in Kahneman’s book (pp. 24–25), so it is not unreasonable to interpret them as his intended meanings. ^3^

Around the edges of attentional research there are indications that attention and effort are distinct (Bruya, 2010). If it is true that attention cannot be identified with effort, it will have important repercussions for cognitive theories moving forward and for practical applications, such as the medical condition of ADHD. We are undertaking our own research program to demonstrate empirically that attention is not identical with effort (Tang and Bruya, 2017). In this paper, we will focus on the theoretical aspects, trying to understand exactly what Kahneman’s influential theory pins down and what is left ambiguous. When faced with ambiguities, we will attempt to give the most plausible reading of the theory and then evaluate evidence in light of that reading.

There are many aspects of effort about which Kahneman is unclear. In fact, in the only review of *Attention and Effort* that we can find, although the reviewer gives a glowing evaluation of many aspects of the book, he says, “Nowhere is effort operationally defined with precision, and indeed the concept is rather elusive” (Moray, 1974). In the sections that follow, we closely examine Kahneman’s theory and put together the strongest interpretation of the theory that we can. These will come in a series of numbered inferences. After making the best case for the theory, we examine it in light of available empirical evidence. Toward the end of the article, we offer our own alternative paradigm, which we plan to expand in future publications. The focus of this article is Kahneman’s paradigm.

## I1. Effort is Cognitive

The first interpretive inference (I1) we can make about Kahneman’s theory of effort is that it distinguishes the mental from the physical. The title of Chapter 2 is “Toward a Theory of Mental Effort.” The mental/physical distinction is now somewhat outdated, however, as most researchers currently refer to the *cognitive*, rather than the *mental*, with the assumption that the cognitive has a physiological basis in the nervous system. “Mental” can be misconstrued as playing on a dualist conception of the spiritual mind as distinct from the physical body. Going forward, we shall refer to cognitive effort rather than mental effort.^4^

## I2. Effort is Objective Not Subjective

The second interpretive inference we can make about Kahneman’s theory of effort is that it distinguishes between the objective and the subjective. Effort as a cognitive process is objective, and the feeling of effort is subjective. Kahneman suggests at one point that he may be referring to effort as subjective (see quotation below), but he appears to understand it largely as objective. This leaves us with the question of whether he views it as objective only or as both objective and subjective. The pertinent passage for the subjective stance is:

“This conception of mental work suggests that time-pressure must be an important determinant of effort. This is a familiar idea in the context of physical exertion: anyone who has tried jogging knows that even a small increase of speed beyond the relatively effortless “natural” speed causes a disproportionate increase in the sense of strain. (p. 25)”

The reference to a sense of strain as an indication of cognitive work is clearly a reference to a subjective feeling of exertion. If Kahneman were interested in studying effort as the feeling of exertion, the obvious method would be to use self-report instruments. Because Kahneman does not use self-report measures or refer to effort again in terms of a subjective feeling, it seems safe to conclude that the subjective feeling is not an integral aspect of his theory of effort. However, the question remains as to the role of subjective feeling in developing an adequate theory of cognitive effort. We shall highlight key unresolved theoretical questions as they arise, in the form of numbered questions.

Q1: Is the subjective feeling of effort significantly distinct from objective effort? If so, what is the role of subjective feeling in developing an adequate theory of cognitive effort?

Because of the lack of discussion of this question in *Attention and Effort*, we shall set it aside.

## I3. Effort is Metabolic Expenditure

What appears to be the key set of passages to understanding what Kahneman means by effort comes early in the book. The first comes in the preface.

While serving as [David Rapaport’s] research assistant for one summer many years ago, I was introduced to the psychoanalytic view of attention as energy. Many years later, having become (as I thought) a rather tough-minded experimental psychologist, I was surprised to discover that my understanding of attention bears the permanent imprint of that encounter. (p. x)

This passage provides us with the tantalizing suggestion that the theory to come will be importantly related to viewing attention as energy. This inference can be further sustained with support from the following extended passage:

A capacity theory of attention [as offered in the present book] provides an alternative to theories which explain man’s limitations by assuming the existence of structural bottlenecks. Instead of such bottlenecks, a capacity theory assumes that there is a general limit on man’s capacity to perform mental work. It also assumes that this limited capacity can be allocated with considerable freedom among concurrent activities. A capacity theory is a theory of how one pays attention to objects and to acts. In the present work, the terms “exert effort” and “invest capacity” will often be used as synonymous for “pay attention.”

Prior to the introduction of a capacity model, it may be useful to briefly consider the question of how a mental activity is to be represented in a cognitive theory. As an example, consider such activities as “recognizing the visual word CAT,” “rehearsing the word BLUE,” or “deciding to press the right-hand key in the display.” Theories of cognitive function usually assume that to each such activity there corresponds a hypothetical structure, and that the activity occurs when the state of the structure is temporarily altered. For example, many theorists would agree that there is a structure corresponding to the word CAT: it has been called a trace, a category state, a dictionary unit, or a logogen. Something happens in that structure whenever the word CAT is presented and recognized. The structure is specific, and its activation depends on the presence of the appropriate specific input.

It is already known that much of the basic sensory analysis of stimuli proceeds in this manner. Thus, there may be one or several neurons in the visual cortex which shift into a characteristic state of activity whenever any conceivable visual stimulus is presented, for example, a corner-shape moving from left to right in a particular region of the retina.

The recognition of specific stimuli by specialized detectors provides an attractive model for a more general theory of the activation of cognitive structures. Indeed, it is tempting to think of the hypothetical structure which “recognizes” the input CAT as basically similar to a corner-detector. In such a system, the appropriate input (from the outside world or from the activity of other neural structures) serves as a key which releases some of the energy contained in the structure and causes it to generate outputs to serve as keys for other structures, and so forth. Because the structures do not share a common source of energy, considerations of overall capacity are not necessary to describe the system. Only the structural connections between the components and the thresholds for the activation of each need to be specified. Structural models of the type illustrated in Figure 1-1 are most easily justified in such a view of information-processing.

Two observations of the present chapter suggest that such a description of information-transfer in man may be inadequate. First, it was noted that momentary variations in the difficulty of what a subject is trying to do are faithfully reflected in variations of his arousal level. There would seem to be little reason for such arousal variations if energy transfer plays no significant role in the system. The second observation was that the ability to perform several mental activities concurrently depends, at least in part, on the effort which each of these activities demands when performed in isolation. The driver who interrupts a conversation to make a turn is an example.

These observations suggest that the completion of a mental activity requires two types of input to the corresponding structure: an information input specific to that structure, and a non-specific input, which may be variously labeled “effort,” “capacity,” or “attention.” To explain man’s limited ability to carry out multiple activities at the same time, a capacity theory assumes that the total amount of attention which can be deployed at any time is limited. (pp. 7–9)

Kahneman identifies his theory as a capacity theory of attention, meaning: (1) attention is not an unlimited resource and (2) attention is a shared resource.

In the above passage, Kahneman begins by describing a theory of cognitive activation and then positively affirms it: “it is already known that much of the basic sensory analysis of stimuli proceeds in this manner” (p. 8). Next, he describes the possible activation of energy from disparate sources in response to specific stimuli but says that these sources, according to descriptions of information-processing theories by others, are not mentioned in the other theories because they need not be. If there are sufficient sources of energy to supply information-processing needs, the sources of energy need not be described in the system since the description is attempting to account for limitations on the system. He then describes two inadequacies of such information-processing theories that do not take energy expenditure into account: (1) variations in arousal suggest variations in energy transfer and are not explained by information-processing alone, and (2) activities make demands that require reallocation of limited resources from competing activities.

Except for one instance later in the book (p. 25), where “energy” appears to be synonymous with “effort,” this is the last time that Kahneman mentions the word “energy” and instead seems to substitute the word “effort.”

Although the use of the term “energy” in the above passage is in reference to other theories, Kahneman uses the notion as a way of criticizing the other theories, suggesting that they lack something that a better theory would not lack. He then goes on to describe a better theory, and so it is natural to infer that his better theory does not lack an account of energy expenditure. Perhaps the reason that he switches from speaking of energy to effort is that his theory is functional rather than physiological. (This would also account for his use of the term “mental.”) Eventually, however, functional theories must be grounded in the actual physiology of the nervous system, and we conclude from the above considerations that the best way to make sense of Kahneman’s theory of attention as effort is to understand effort as a metabolic expenditure that occurs inside the brain’s nerve cells.

It is acceptable to us to think of cognitive work as involving metabolic expenditure at the level of the nerve cell. An important question arises from this interpretation:

Q2. Is effort, as metabolic expenditure, applicable to only a special case of cognitive activity, or is it applicable to all cognitive activity?

We shall discuss a possible answer to this question below.

## I4. Effort is Attention

On page 4, Kahneman says:

The present work contends that intensive aspects of attention must be considered in dealing with voluntary as well as with involuntary selection. For this integration to be possible, however, the intensive aspect of attention must be distinguished from the more inclusive concept of arousal. Thus, the schoolboy who pays attention is not merely wide awake, activated by his teacher’s voice. He is performing work, expending his limited resources, and the more attention he pays, the harder he works. The example suggests that the intensive aspect of attention corresponds to effort rather than to mere wakefulness. In its physiological manifestations effort is a special case of arousal, but there is a difference between effort and other varieties of arousal, such as those produced by drugs or by loud noises: the effort that a subject invests at any one time corresponds to what he is doing, rather than to what is happening to him.

The identification of attention with effort suggests a reinterpretation of the correlation between arousal and involuntary attention. Novel and surprising stimuli which spontaneously attract attention also require a greater effort of processing than do more familiar stimuli. The surge of arousal that follows a novel stimulus represents, at least in part, a surge of mental effort. In this view, voluntary attention is an exertion of effort in activities which are selected by current plans and intentions. Involuntary attention is an exertion of effort in activities which are selected by more enduring dispositions.

One key theoretical claim in this passage is the identification of attention and effort. When two things are claimed to be identical, the claim is that they are one thing and not two—they are logically interchangeable. Thus, according to Kahneman’s theory, every instance of attention is an instance of effort, and every instance of effort is an instance of attention.

This identification of effort and attention appears to resolve Q2—effort is a special case of cognitive activity, it is attention. However, attention is not understood by Kahneman as a specific case of cognitive activity. A fundamental part of Kahneman’s capacity theory is that effort-attention^5^ is a shared resource. For this reason, it cannot be confined to a single area of the brain or to a particular anatomical network.

Since the publication of Kahneman’s book, Posner and Dehaene (1994), Fossella et al. (2002), and Fan et al. (2005) have shown evidence for distinct attentional networks in the brain. Kahneman could accept an attentional network theory only by viewing it as a kind of pipeline channeling resources or as a network facilitating resource utilization, rather than as a specific site or activity of resource utilization. Because nerves participate in activating metabolic energy rather than channeling it, the best way to make sense of Kahneman’s idea physiologically is to postulate that effort-attention is a shared resource in terms of blood flow and energy activation within cells, activated by neural networks. In light of what we know about brain physiology, only blood flow can act as a shared energy resource in the brain. The answer to Q2, then, must be that effort-attention is not applicable to a specific case of cognitive activity but applies potentially to all cognitive activity reached by an activating network.

If the identity claim is true; however, it raises a further question:

Q3. Since the brain is constantly active, what distinguishes effort-attention from other cognitive metabolic expenditure? Is it a functional difference (effort-attention does something that other kinds of cognition do not?), a subjective difference (effort-attention is metabolic expenditure that is conscious), a difference in magnitude (effort-attention is a large metabolic expenditure that has crossed a threshold of magnitude), or something else? We’ll take these one at a time.
  Q3.1 Is effort-attention functionally distinct? Intuitively, one would think that attention must be a distinct case of cognitive activity, but Kahneman’s whole point in identifying attention with effort seems to be that effort-attention is nothing more than the investment of resources in an occurrent cognitive activity, and that activity could be anything. The answer to this question, then, must be no.
  Q3.2 Is effort-attention distinct in virtue of being conscious? Kahneman does not discuss consciousness, nor does he imply it. Therefore, we have no choice but to set this question aside.
  Q3.3 Is effort-attention distinguished from other metabolic brain activity in terms of magnitude? It could be that what brings an occurrent cognitive activity into attention is that there is an increase in metabolic magnitude. Metabolic activity below a certain magnitude is outside of attention, and metabolic activity above a certain magnitude is attentional. This concept is also not discussed by Kahneman, but it is implied in his many discussions of the quantification of effort-attention. Kahneman relies largely on pupillometry as an index of effort-attention, tying it to sympathetic arousal, which can be taken as the mobilization of metabolic resources. We will discuss this further below.

In pursuing the best reading of Kahneman’s theory that effort is attention, we conclude from the above that: effort-attention is an increase in metabolic activity in the brain.

## I5. Effort-Attention is an Increase in Metabolic Activity in the Brain

This answer to Q3 raises another question:

“Q4: What does it mean that there is an increase in metabolic activity?”

Is that an increase from baseline? If so, what is the baseline? Is it an increase relative to activity immediately prior? Is it an increase of activity in this area relative to other specific areas? Is it an increase in this area relative to an average of activity in this area over some period of time in the past? These particular clarificatory questions are not answerable from the book, but they all lead in the same direction: effort-attention is an increase in metabolic activity in the brain that has crossed a threshold of magnitude.

## I6. Effort-Attention is an Increase in Metabolic Activity in the Brain That Has Crossed a Threshold of Magnitude

We can call the crossing of this threshold *superliminal* (in a generic, not Freudian, sense). Thus, effort-attention is a superliminal allocation of metabolic resources applied to cognitive tasks.

In order to get a detailed understand understanding of effort-attention according to Kahneman’s theory, one would have to measure the metabolic activity of cognitive tasks. Such a measurement was not available to Kahneman. As a proxy, he used physiological indications of autonomic activity, especially pupil dilation. Pupil dilation has long been known to be activated by sympathetic nerve fibers of the superior cervical ganglion (Hess, 1972). In the passage above, Kahneman says: “in its physiological manifestations, effort is a special case of arousal.” Since arousal is defined by Kahneman as sympathetic dominance (p. 18), Kahneman’s understanding of effort-attention is: effort-attention is a special case of sympathetic dominance of the autonomic nervous system.

## I7. Effort-Attention is a Special Case of Sympathetic Dominance of the Autonomic Nervous System

A question immediately arises:

Q5: What distinguishes effort-attention from other instances of arousal (sympathetic dominance)?

Kahneman says that it is not difficult for a researcher to tell the difference between arousal as effort-attention and other “contaminating factors” (p. 22), but he does not provide any kind of objective measure, leaving Q5 unanswered. In what follows, we shall pursue these two considerations in more detail: is it possible to distinguish effort-attention from other forms of sympathetic arousal, and is it possible for there to be effort-attention absent sympathetic arousal?

## I8. Effort-Attention is a Special Case of Arousal

Kahneman attempts to distinguish arousal-as-effort-attention from other kinds of arousal, as follows.

### Arousal as Muscular Strain

Kahneman (pp. 22–23) cites a study in which subjects performed the same mental task under “say” and “thought” conditions, and controlling for muscular movements of the “say” condition, the pupillary results were essentially the same. Kahneman concludes that muscle strain does not account for pupil dilation, but effort-attention does.

### Arousal as Anxiety

Kahneman (pp. 23–24) cites studies that attempt to tease out the differences between arousal as anxiety and arousal as effort-attention. He says that if arousal were due to anxiety in a task situation, anxiety (and therefore pupil dilation) would occur in anticipation of failure and after failure occurs. However, he cites studies showing that pupil dilation is largest during the performance of a task and larger for success not failure.

### Evidence Against Effort-Attention as a Kind of Arousal

Kahneman’s work seems to have cemented the theory that pupil dilations track cognitive effort, not arousal broadly.^6^ However, Geva et al. (2013) performed a series of experiments in which subjects engaged in the Attention Network Test while monitoring autonomic responses, including pupil dilation. Geva et al. (2013) note that pupil dilations subside with practice in incongruent executive attention tasks. They conclude, “These marked differences in effect size may point to a specific role for [executive control pupil response (Pe)] in investing effort in monitoring, such that as the level of practice increases, the amplitude of Pe decreases, signifying that less effort is needed to maintain near-perfect accuracy performance in high-load tasks that entail a risk of errors” (p. 7). Although, Geva et al. (2013) suggest that both effort and attention decrease, this can’t be true under the definition established above. In the experiment, the load remains high, and the demands are being met. Thus, there must still be high metabolic expenditure to meet the demands. If effort-attention is metabolic activity and metabolic activity remains high, then effort-attention cannot simultaneously decrease. Either effort is not attention or effort-attention is not superliminal metabolic activity.^7^

Geva et al. (2013) perpetuate the confusion about effort by stating that monitoring, itself, is effortful. Is it the cognitive activity in response to load that is effortful or the monitoring that is effortful? These two things are distinct. When effort is not clearly operationalized, it has a tendency to drift in meaning to fit the needs of a theory.

Servan-Schreiber et al. (1990) modeled the locus ceruleus norepinephrine (LC-NE) system at the level of individual neurons and hypothesized that LC has the function of adaptive gain modulation—meaning that in response to increased demands it heightens arousal in order to enhance sensitivity to some stimuli, inhibits sensitivity to other stimuli, and readies appropriate responses. Aston-Jones and Cohen (2005) built on this work, studying the relationship among LC-NE, orbitofrontal cortex (OFC) and anterior cingulate cortex (ACC), finding that LC-NE is signaled by calculations from the OFC and ACC. In other words, pupil dilation, rather than signaling the expenditure of resources, signals a readiness to expend resources. We contend that this is more likely to be what attention actually is, and although it requires some resource expenditure, as all neural activity does, it is not resource expenditure in response to load but rather is the readying of response to load. The adaptive gain theory has been further explored and confirmed in studies by Gilzenrat et al. (2010), Jepma and Nieuwenhuis (2011), Nieuwenhuis et al. (2011), and Joshi et al. (2016). From this perspective, it appears that attention is not effort but a combination of enhanced sensitivity and responsiveness within a specific context.

Therefore, I4–I7 are not borne out by the evidence. Effort, under the construal above and in light of recent evidence, cannot be attention. However, under the adaptive gain theory, it appears that attention (though not effort) can still be closely associated with arousal and can be indexed by pupil dilation.

In Kahneman’s theory, because effort-attention is indexed by sympathetic arousal, it is safe to infer: effort-attention cannot occur without sympathetic dominance.

## I9. Effort-Attention Cannot Occur Without Sympathetic Dominance

### Kahneman’s Case for the Connection Between Effort-Attention and Sympathetic Dominance

Kahneman (pp. 29–33) considers the case of directional fractionation, when some autonomic signals show sympathetic arousal and some do not. He cites studies in which pupils dilate but heart rate slows. These cases, Kahneman shows, occur in an inhibitory state of anticipation of stimuli, as when viewing interesting pictures. They do not occur, he says, during task performance, when all sympathetic arousal signals align to show sympathetic dominance.

However, Kahneman cites a study by Elliott (1969) in which heart rate slows during the Stroop task. Ignoring the fact that performing the Stroop task is a clear case of task performance, Kahneman attributes the directional fractionation to an inhibitory state, when the subject is disrupting response conflict by actively reading the Stroop word.

It is important to notice Kahneman’s sleight-of-hand here. He begins by inquiring whether directional fractionation is a counter-example to his claim that attention-effort occurs only under sympathetic dominance. He ends by distinguishing three distinct kinds of arousal. One of these is the inhibitory, fractionated kind, associated, he says, with “alertness” (p. 33). The second is a “pattern of relaxed acceptance” (p. 33), which he classifies closely with the first kind. The third kind is “generalized sympathetic dominance” (p. 33), which occurs during “problem-solving” (p. 33). Suddenly, the notion of attention is narrowed to only the kind that is effortful and occurs under sympathetic dominance. Instead of demonstrating that effort-attention occurs only under sympathetic dominance by showing that there are no cases of attention under directional fractionation, Kahneman redefines effort-attention so that only attention that occurs under sympathetic dominance counts as effort-attention. He ignores instances of attention that happen under directional fractionation. In other words, attention is attention only when it is effortful. If it is not effortful, it doesn’t count as attention. This demonstrates the danger of not first defining terms. Why is the inhibitory condition not a case of attention?

### Recent Evidence for Attention Without Sympathetic Dominance

We do not dispute that attention can arise and function under sympathetic dominance. As we argue above, however, it is important to distinguish effort from attention.

If it were true that effort and attention were identical and that sympathetic dominance was a necessary signal of effort and attention, it would be impossible for attention to occur in the absence of sympathetic dominance, especially during parasympathetic dominance. And yet, there are now a number of studies that describe attention co-varying with parasympathetic dominance.

### Meditation

Meditation is an activity in which a person intentionally focuses attention on a single item or domain internally or externally, such as a mandala, the breath, or a thought or feeling of compassion. By definition, meditation is an attentional activity. Kubota et al. (2001) induced a state of what they called *relaxed concentration* through breath meditation while monitoring EEG and ECG activity. Sustained frontal theta rhythm is associated with high attention in meditation (Aftanas and Golocheikine, 2001; Lagopoulos et al., 2009) and when subjects achieved sustained frontal theta rhythm, Kubota et al. (2001) found a simultaneous increase of heart rate variability (HRV) in subjects, a classic sign of parasympathetic dominance. They also found that sympathetic tone was negatively correlated with Fz theta rhythm. ^8^ Takahashi et al. (2005) and Tang et al. (2009) found a positive correlation between HRV and theta activity during meditation. ^9^ In addition to HRV, Tang et al. (2009) included measures of heart rate, skin conductance, belly amplitude, and respiratory rate, all of which showed increased signs of parasympathetic dominance during meditation. Tang et al. (2009) also included brain imaging in their studies of meditation and found that subgenual and adjacent ventral ACC activity correlated with the above physiological signs of parasympathetic dominance. Wu and Lo (2008) did not measure EEG activity but also found increased HRV during meditation. ^10^ In addition, Amihai and Kozhevnikov (2014) found elevated HRV during *vipassana* meditation. ^11^ Butler et al. (2006) and Bornemann et al. (2016) reported increased HRV during distinct mindful self-regulation exercises. ^12^ The results of the above studies combine to show that attention can occur under parasympathetic dominance. Although some meditation studies have also shown signs of sympathetic dominance in distinct kinds of meditation (Amihai and Kozhevnikov, 2014) or evidence of directional fractionation, the evidence cited above is a profound challenge to the notion that attention necessarily occurs under sympathetic dominance.

## Attention and Intention

We propose that the base of the problem in *Attention and Effort* is twofold. First, Kahneman assumes a long-standing belief in Western culture that effort is valuable in and of itself (Kant, [1785] 1959; Weber, 1930). Because of this assumption, he adopts the high-effort, high-attention schoolboy-in-class as the model of attention, ignoring other obvious instances of attention, as in low-effort, high-attention game-playing. He also ignores contradictory evidence. Second, he restricts his available evidence almost entirely to laboratory studies of high-effort, high-attention activities, rarely considering in-laboratory activities of low-effort, high-attention or more ecologically valid attentional activities.

We believe that in order to be comprehensive in building cognitive models, one must take a fundamentally biological approach to cognition, recognizing the human being as a product of natural selection, guided by an intentional perspective on the world (Brentano, [1874] 1973; Dewey, 1958; Dennett, 1987; Freeman, 2000). This intentional perspective commonly involves attentional, goal-directed behavior in an environment of many, constantly changing demands. In order to cope with these demands, a person can sometimes limit one’s focus to a narrow task at hand. Other times, it is better to attend to a broader array of stimuli. These distinct kinds of attention have been called *exploitation* and *exploration*, respectively (Kaelbling et al., 1996; Usher et al., 1999; Doya, 2008). Both are kinds of attention. There is evidence that the LC-NE system regulates both exploration and exploitation (Aston-Jones and Cohen, 2005; Jepma and Nieuwenhuis, 2011), resulting in sympathetic dominance and pupil dilation for exploitation. There is little evidence, outside of the LC-NE system, about the underlying physiology of the exploration mode of attention. We suggest that it is modulated by a network involving the ACC, insula, and striatum and characterized by parasympathetic dominance (Tang et al., 2012).

## Conclusion

In this article, we examined Kahneman’s argument that attention is effort. In giving the best possible reading, we isolated the following relevant inferences that can be made from his argument:

I1. Effort is cognitive.
I2. Effort is objective not subjective.
I3. Effort is metabolic expenditure.
I4. Effort is attention.
I5. Effort-attention is an increase in metabolic activity in the brain.
I6. Effort-attention is an increase in metabolic activity in the brain that has crossed a threshold of magnitude.
I7: Effort-attention is a special case of sympathetic dominance of the autonomic nervous system.
I8. Effort-attention is a special case of arousal.
I9. Effort-attention cannot occur without sympathetic dominance.

We found that the weak link in Kahneman’s argument is that the term “effort” is insufficiently defined, and that the step from I3 to I4 is not warranted, toppling all the inferences that follow. We do not disagree that attention may be associated with sympathetic dominance, but recent evidence shows that this is not a necessary link as the identification of attention and effort would seem to entail. Rather, there appear to be two modes of attention. One mode of attention is associated with sympathetic dominance and adaptive gain modulation (which is distinct from effort, *per se*) to handle the demands of cognitive tasks. The other mode of attention is associated with parasympathetic dominance and may be experienced as effortless.

What is effort? We propose that Kahneman is correct that objective cognitive effort can occur with attention under sympathetic dominance. However, attention can also be achieved without sympathetic dominance, and, of course, sympathetic dominance can occur in the absence of attention. Can high objective cognitive effort occur without high attention? That depends on what one means by high objective cognitive effort. A clear delineation of objective cognitive effort deserves further conceptual analysis (see, Richter and Wright, 2014, for a summary and examples of recent attempts in this direction).

## Implications for Further Research

In the course of our analysis of Kahneman’s argument, a number of questions arose, of which the following deserve further study.

(1) Is the subjective feeling of cognitive effort significantly distinct from objective cognitive effort? If so, what is the role of subjective feeling in developing an adequate theory of cognitive effort? See Bruya (2010) and Robinson and Morsella (2014) for recent discussions.

(2) What is the relationship, if any, among effort, attention, and metabolic expenditure in the brain? See Fairclough and Houston (2004), Noakes (2012), and Inzlicht and Marcora (2016) for recent discussions.

Developing adequately operationalized concepts of effort and attention will require interdisciplinary cooperation that recognizes the entire range of human cognition and action. Not only should it include laboratory studies of directed high-attention tasks but should also include high-attention activities in ecologically valid circumstances. High-attention activities are often endogenously motivated, and insofar as motivation is a component of the perception-action cycle it should not be neglected (see, for example, Kruglanski et al., 2012). Studied tasks should include high-attention tasks that may not necessarily be experienced as effortful, such as various forms of meditation, as well as games (including video games), hobbies, crafts, and sports. Assessing the effort and attention within this wide range of activities will require expertise measuring various phenomena in an equally wide range of disciplines, such as neurology, biochemistry, genetics, kinesiology, and cardiology. Terminology must also be carefully delineated. Effort, for example, must be distinguished from load, and not only are there both cognitive and physical dimensions of both effort and load, there are also subjective and objective dimensions. How can one accurately measure objective and subjective cognitive load and the objective and subjective cognitive effort required to “displace” that load? Are there units for measuring objective cognitive load and objective cognitive effort? Is it even theoretically possible to find matching units such that we can say: *x* units of cognitive effort are required to “displace” *x* units of cognitive load? At the moment of “displacement,” what exactly occurs at the neurological and metabolic levels? Must we also distinguish, as in physics, between resistance force and effort force? These are the kinds of difficult questions that remain to be answered.

## Author Contributions

All authors listed have made a substantial, direct and intellectual contribution to the work, and approved it for publication.

## Conflict of Interest Statement

The authors declare that the research was conducted in the absence of any commercial or financial relationships that could be construed as a potential conflict of interest.

## Footnotes

1 We are not suggesting that Kahneman’s purpose in writing the book was to identify attention with effort. It is, however, a key, and influential, step in his overall argument.

2 See, for example, Martin Sarter on attentional effort as cognitive incentive (Sarter et al., 2006), Stanislas Dehaene on attentional effort in a global workspace (Dehaene et al., 2001), and Christopher Wickens on the ergonomics of attention and effort (Wickens and McCarley, 2007).

3 Moray (1974), in a review of the book, says, “At times (‘effort’) seems to refer to the subjective experience one has when trying to attend” (p. 698).

4 We assume a general reductionist desideratum in the cognitive sciences. This seems to be a safe assumption when evaluating Kahneman’s work because of his reliance on physiological markers in developing his theory. We evaluate the empirical evidence that he adduces in support of his theory and consider more recent evidence.

5 Because Kahneman identifies attention and effort, wherever one is mentioned, the other can be assumed. To make this clear, we henceforth refer to both terms as *effort-attention* in reference to Kahneman’s theory.

6 Just one example of the widespread acceptance of this theory can be seen in the opening line of the abstract of Hoeks and Levelt (1993), which says, “It has long been known that the pupil dilates as a consequence of attentional effort.”

7 Above, we concluded, according to Kahneman’s theory, that effort-attention is not just metabolic activity but metabolic activity that crossed a threshold of magnitude. The only way to maintain this definition, given Geva et al. (2013) results, is to posit a variable threshold of magnitude, which would lead back to the unanswered questions following Q4.

8 Kubota et al. (2001) used Toichi’s Cardiac Sympathetic Index (CSI) to calculate sympathetic tone and Toichi’s Cardiac Vagal Index (CVI) to calculate parasympathetic tone (Toichi et al., 1997) from ECG data.

9 Takahashi et al. (2005) and Tang et al. (2009) both relied on frequency-domain analysis of the high frequency (HF) spectrum of ECG data as their measure of HRV.

10 Wu and Lo (2008) used HF analysis to assess HRV.

11 Amihai and Kozhevnikov (2014) used HF analysis to assess HRV.

12 Butler et al. (2006) had subjects focus on trying to “look on the bright side” and “find anything positive you can in the film or the conversation” during a conversation about elicited emotions. Respiratory sinus arrhythmia (RSA) substituted for HRV and was measured using a custom algorithm based on HF. Bornemann et al. (2016) had subjects engage in a biofeedback exercise designed to upregulate vagal tone. They used a custom algorithm based on HF to assess HRV.
